# Supplementary material for: Locomotion of Ants Walking up Slippery Slopes of Granular Materials
Source: Integr Org Biol. 2019 Sep 13;1(1):obz020. doi: 10.1093/iob/obz020 (PMC7671155; doi:10.1093/iob/obz020)
Supplement: obz020_Supplementary_Data [file obz020_supplementary_data.zip › Final IOB-2019-011.pdf]

Locomotion of ants walking up slippery slopes of granular materials

Antoine Humeau[1], Miguel Piñeirua[1], Jérôme Crassous[2], and Jérôme Casas[1,3]

[1]Institut de Recherche sur la Biologie de l'Insecte, UMR 7261 CNRS - Université François - Rabelais, 37200 Tours, France.
[2]Université Rennes 1, Institut de Physique de Rennes (UMR UR1-CNRS 6251),Campus de Beaulieu, F- 35042 Rennes, France.
[3]Institut Universitaire de France.
Corresponding author: jerome.casas@univ-tours.fr

July 3, 2019

Abstract

Many insects encounter locomotory difficulties in walking up sand inclines. This is masterfully exploited by some species for building traps from which prey are rarely able to escape, as the antlion and its deadly pit. The aim of this work is to tear apart the relative roles of granular material properties and slope steepness on the insect leg kinematics, gait patterns and locomotory stability. For this, we used factorial manipulative experiments with different granular media inclines and the ant *Aphenogaster subterranea*. Our results show that its locomotion is similar on granular and solid media, while for granular inclined slopes we observe a loss of stability followed by a gait pattern transition from tripod to metachronal. This implies that neither the discrete nature nor the roughness properties of sand alone are sufficient to explain the struggling of ants on sandy slopes: the interaction between sand properties and slope are key. We define an abnormality index that allows us to quantify the locomotory difficulties of insects walking up a granular incline. The probability of its occurrence reveals the local slipping of the granular media as a consequence of the pressure exerted by the ant's legs. Our findings can be extended to other models presenting locomotory difficulties for insects, such as slippery walls of urns of pitcher plants. How small arthropods walking on granular and brittle materials solve their unique stability trade-off will require a thorough understanding of the transfer of energy from leg to substrate at the particle level.

1 Introduction

Through millions of years of evolution, insects have succeeded at conquering the whole earth, from the equator to the polar regions [1, 2]. They are therefore constrained to move on a large diversity of media to survive. For example, many arthropods such as mites, aphids or ladybirds that walk on plant leaves [3], have to deal with complex and unpredictable leaf oscillations, deformations and rotations due to wind [4]. Some predators also exploit the use of different kind of surfaces specifically designed to promote locomotive difficulties for pray. Two remarkable examples of these deadly techniques are the slippery peristome of carnivorous plants [5] as well as the sticky strings in spider webs [6].

Legged locomotion encompasses thus a large variety of environments. Recent studies have adressed the problem of insect locomotion in difficult ground environments, such as, for example, the way cockroaches move within grass, logs and fungi [7, 8]. Nonetheless, insect walking in and

on soils has been poorly studied despite the fact that this habitat is heavily populated by a whole range of arthropod groups, and since a long time [9]. Soils are highly heterogeneous media which can vary greatly in cohesion, particle size, amount of colloids, etc. Among the different types of soils, granular materials like sand are of particular interest due to their presence in a large variety of environments like deserts, dunes, arid regions, sea shores or river banks. All of the latter are ecosystems inhabited by a high diversity of insects, which have adapted their locomotive patterns in order to enhance their mobility. Such is the case of *Cataglyphis* ants or *Pachysoma* beetles, that efficiently walk on dunes and sandy flat land [10]. Some ants even build conical sand structures at the entrance of their nest and walk normally on their slope [11, 12].

At the same time, some predators, as for example antlions, exploit the physical properties of granular media in order to catch prey. The way antlions capture their prey has been the focus of many general observational works since over a century (see [13] and references therein) but only a handful manipulative works on the physics of the pit have been conducted [14, 15]. Antlion pits consist of a cone dug in the sand; the larva sits at the bottom, waiting for the prey to fall down. The pit is built with a slope very close to a critical angle, that defines the steepest slope not leading to an avalanche [16, 17]. These studies have shown that a pit functions best when the sand is dry, the slope is steep and the sand grains are of small size (in the range of 100 to 500 μm).

However, the physics of the surface characteristics of sand slopes impeding insects to move up and out of the pit is still unknown. A recent study [18] has shown that the friction solid coefficient in sandy slopes near the avalanche angle is pressure dependent. While heavy objects tend to generate footprintlike deformations which stabilize them on the slopes, light objects do not disturb the sandy surfaces and remain also stable. However, for intermediate weights, the surface deformations lead to a sliding phase with loss of stability. Could this explain the fact that some insects escape without difficulty while others do struggle a lot? The exploration of this new hypothesis appears to be crucial to the understanding of the physical mechanisms of locomotory inability of insects to escape out of a sand pit, and more generally to move up a sand slope. In fact, despite the many studies on antlions and their prey, we have no information on several relevant parameters for their interaction, such as the prey leg kinematics, the compaction of sand in the pit and the degree of humidity of the sand (see for example [19]). In order to provide an appropriate basis for explaining the physics of the locomotory difficulties of insects in antlion pits, and more generally on sand slopes, more controlled and manipulative experiments including precise descriptions of the granular media and insect movements are required.

In the first part of this work we compare, by means of high-speed video recordings, the locomotion of the ant *Aphaenogaster subterranea* walking in seven different conditions : solid or granular media, flat or inclined, smooth or rough surfaces and in the antlion pit. We focused on this ant species as biological model because ants are the main taxon identified among antlion prey, accounting for between 35 and 70% of all diets (see [13]). This species is also known to be the archetype of an average ant which gets trapped in the ant lion pit. In this first part of the manuscript, we show that neither the slope, nor the granular medium effects alone can explain the observed changes in the ants gait and kinematics. This results lead to the second part of this work, in which we study the interaction between granular media properties and plane inclination. In order to explore the interaction between granular media and slope, we vary the inclination of the plane and study the locomotion of the ants around the angle of avalanche, the threshold at which a granular material transits from a solid into a fluid phase. Such phase change occurs at a precise angle value for a given particle geometry, thus requiring a series of highly controlled experiments.

2 Materials and Methods

2.1 Experiments with natural sand

Biological material

Workers of the woodland ant *Aphaenogaster subterranea* (Latreille 1798) (Hymenoptera: Formicidae) were obtained from two colonies located in the Grandmont Parc in Tours (France, 47.354^o N, 0.704^o E). The mean weight of the collected individuals used throughout the experiments was 1.74 ± 0.4 mg, with a typical length varying between 3 to 4.7 mm. We collected second and third-instar larvae of antlion *Euroleon nostras* (Geoffroy 1795) (Neuroptera: Myrmeleontidae) from the same site and maintained them in the laboratory.

Experimental design

Experiments were designed in order to vary : (i) the type of substrate, solid or granular, (ii) the slope, flat or inclined and (iii) the roughness, smooth or rough (Table 1). A treatment refers to a complete or partial combination of the three variables. For example, there were four inclined treatments irrespective of substrate and roughness, and two solid-flat treatments irrespective of roughness. By definition, there was no granular-smooth treatment, neither flat nor inclined. The six tested treatments (T1-T6) represent all possible entries of the design matrix. The pit built by an antlion (T7) represents an additional treatment used to compare our artificial experiments with natural conditions. The experiments were conducted in a 337 x 164 x 321 mm terrarium. We used a glass plate for the solid-smooth treatments (T1 & T2). The solid-rough treatments (T3 & T4) were constructed by gluing Fontainebleau sand (of typical grain size between 100 and 315 μm) to the glass plate. We therefore assume that there were no loose grains. The same Fontainebleau sand was used for the granular treatments (T5-T7). For these, sand was poured into the terrarium to a depth of about 6 cm with a funnel. To ensure that the preparation was flat but not compacted, the funnel was moved around the entire surface of the terrarium while pouring. In the granular-inclined treatment (T6), the whole terrarium was inclined to produce an artificial granular slope, up to an angle just below the avalanche threshold. The antlion pits used in T7 measured between 39 and 65 mm in diameter at the outer rim at surface level and 10 and 18 mm in depth. The mean slope angles (measured with respect to the horizontal) in the inclined treatments were 36.1° for the solid-inclined smooth treatment (T2), 36.3° for the solid-inclined-rough treatment (T4), 28.5° for the granular-inclined-rough (T6), and 28.6° ± 1.4° (only treatment for which the s.e. is higher than 0.1) for the pit (T7). This pit angle is small compared to other works where angles of $\sim$ 37° [16, 20] were measured. We have however no good explanations for this difference.

Films

We placed between 10 and 20 ants in the terrarium on the day of capture and allowed them to move freely. Each filmed ant was removed, killed and weighed to the nearest 0.1mg (Table 1). For the pit treatment (T7), we transferred an antlion larva to the terrarium at least one day before filming. Ants and antlions were used only once. The camera, a *Phantom V9.1* with a *Sigma* 50 mm F 2.8 DG Macro, was positioned perpendicular to the horizontal plane and filmed from above, at 100 frames per second. The image definition was 1632 x 1200 pixels. The image encompassed the pit in the pit treatment.

Data acquisition

The positions of the ant and its tarsi were estimated for each frame with the *ImageJ* software. The position of the ant was represented by the centroid of its body, i.e. the ant without its legs

ID	Treatement	N	Film duration (min)
T1	solid-flat-smooth	11	2.19 [0.85-4.25]
T2	solid-inclined-smooth	12	2.05 [1.11-4.84]
T3	solid-flat-rough	10	1.33 [0.63-2.67]
T4	solid-inclined-rough	12	1.60 [0.72-4.77]
T5	granular-flat-rough	11	1.14 [0.80-1.78]
T6	granular-inclined-rough	9	10.29 [2.58-18.24]
T7	pit	11	11.85 [1.60-18.24]

Table 1: Experimental set-up and film properties for the seven treatments. The values shown are the mean [minimum - maximum]. N is the number of used ants. The duration of films in the two granular inclined treatments were longer than in the other five treatments because ants walked with difficulty, thereby staying longer in the camera's field.

and antennae (see Figure 1-E). The body was selected by a semi-automatic method applied to all or parts of each film, with the same set of values (a detailed description of the method can be found in Appendix A.1). The positions of the tarsi were identified by eye, by defining the center of a circle fitting the extremity of a tarsus. This method gave a more accurate estimation of the position of the tarsi than the selection of a single point. These points were used in all analyses that required the recording of the position of legs. The extent of a leg in contact with sand is highly variable; it is at least the tip of the tarsus and never more than the entire tarsus. All measurements were adjusted by means of the spatial calibration presented in Appendix A.2.

Data analysis

In the inclined treatments (T2,T4,T6,T7), we kept only ants which walked mainly in the upward direction. An ant was considered walking upwards for positive differences between the ending and starting positions in the y direction. Ant movements were analysed in two reference frames, the world and the body frames [21]. The "world frame" was fixed for each film and its origin was defined at bottom left corner of the image. The "body frame" was defined in order to follow the ant's movements. Its origin was taken to be the centroid of the ant's body. The origin of the body frame had therefore different positions in the world frame over time.

In the world frame, we compared the speed of the ants between treatments, as an approximation for the ease of movement. The instantaneous speed of an ant at time t was estimated from the total displacement of the ant's centroid between times $t-1$ and $t+1$, corresponding to a 0.02 s interval. Leg movements in the world frame were analysed by measuring five complementary variables: stride period, duty factor, stride length, swing length and slip length, as shown in Figure 1-A. A leg can be at any moment in a "stance state" or in a "swing state". These states are instantaneous descriptors. The stance state is defined by the leg making contact with the surface of the medium. The leg is otherwise considered to be in the swing state.The slip length is a synonym of a "stance length" (the contact point of the leg with the substrate varies as the leg slips). It has a positive value only in the case the leg is slipping, and is null otherwise. The state of each leg, in the swing or stance phase, was determined by eye, frame-by-frame. A leg stride is defined as the succession of a stance and a swing phases. The length of stride is the distance traveled by a leg during a stride. Finally, the duty factor is the percentage of a stride composed of the stance phase.

To easily compare the treatments, we used one value for each ant and each variable, and use the estimated mean of the data (Table 2). This choice implied that each ant had the same weight in the analyses, independently of the duration of the film. For each variable, we therefore fitted a probability density function (pdf) for each ant, pooling the data for the six legs. We

#	Treatement	Speed (mm/s)	Period (s)	Duty factor(%)	Length (mm)		
					stride	swing	stance
1	solid-flat-smooth (sfs)	19.9±2.4	0.22±0.03	66±1	3.7±0.1	3.4±0.1	0.2±0.1
2	solid-inclined-smooth (sis)	19.2±1.9	0.22±0.03	69±1	3.5±0.1	3.2±0.1	0.2±0.0
3	solid-flat-rough (sfr)	25.6±2.4	0.16±0.01	63±1	3.7±0.1	3.4±0.1	0.2±0.0
4	solid-inclined-rough (sir)	26.5±2.4	0.16±0.02	62±1	3.4±0.1	3.1±0.1	0.3±0.0
5	granular-flat-rough (gfr)	22.7±1.1	0.16±0.01	69±1	3.7±0.1	3.3±0.1	0.4±0.0
6	granular-inclined-rough (gir)	5.7±1.1	0.47±0.08	80±1	1.9±0.2	2.0±0.1	0.7±0.1
7	pit	6.5±1.0	0.39±0.04	80±1	1.5±0.2	2.0±0.1	1.3±0.1

Table 2: Ant kinematics in the world frame for the seven treatments. The values are means ± standard errors.

fitted different distributions by moment-matching estimation, using the "fitdist" function of the "fitdistrplus" package in the R environment (Delignette-Muller et al. 2003, R core Team 2013). A single pdf was eventually chosen for each variable, on the basis of graphical explorations of the fits and the results of Kolmogorov-Smirnov tests. For the variables speed, period, stride length, swing length and slip length, we tested normal, log-normal and gamma distributions. The log-normal distribution was the best for the variable period, whereas the gamma distribution was the best for speed, stride length, swing length and slip length. For the duty factor (a proportion), we tested normal and beta distributions and found that the beta distribution was the best.

In the body frame, we only analysed the leg trajectories in the antero-posterior axis, reducing the movement to one dimension for simplicity. We focused on this axis because it represents the axis of forward and backward movements. The axis was defined positive towards the head (see Figure 1-E). The beginning of the stance was used as the beginning of the stride. Each stride was firstly normalized by its duration, and then, for each ant, all normalized strides were grouped by a local smoothing regression ("loess" function in R, with default parameters), separately for front, middle and hind legs. Then, the extension was computed on the basis of the extreme positions of legs during a stride. The extension of a leg during a stride was then compared between the different treatments.

We also analysed the inter-leg locomotory patterns, that is the relationships between the states of legs, thereafter named "gait" (for an example on different gait patterns see Figures 1-B and 1-C). We grouped gaits by counting the number of legs in contact with the ground. The relative importance of the different gaits observed in each treatement was estimated as the average ratio of time spent performing a given gait with respect to the total duration of each experiment.

We finally estimated the surface of sand in movement under a leg during the stance phase in the pit treatment (T7). We estimated this value once per ant for one stance of a middle leg for which the front and hind legs did not interfere with the sand movements caused by the middle leg only. This pair of legs was chosen because the body often obscured sand movement caused by the front legs and because the hind legs were the least mobile. We counted the numbers of grains that moved over the longest (in vertical plane) and the widest (in horizontal plane) distances, leading to a rectangle of moving grains. This measure overestimates the actual number of grains that moved because the real surface is more like an ellipse than a rectangle.

2.2 Experiments with model sand (glass beads)

While the first series of experiments was filmed at a short distance in order to make visual recognition of individual sand grains feasible and to have precise estimation of leg kinematics, the second series of experiments was filmed from a much larger distance and focused on global aspects of locomotion in the world frame.

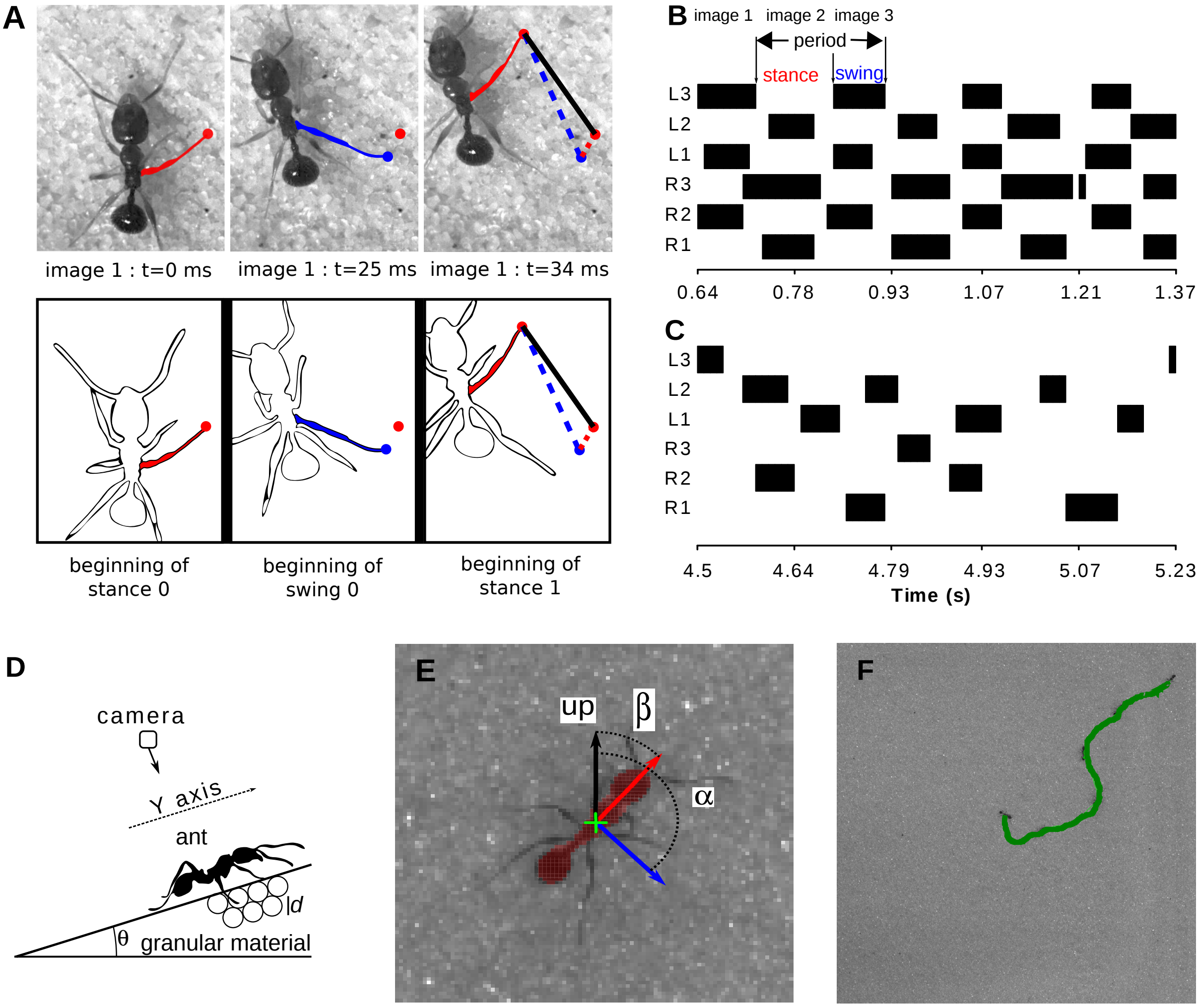

Figure 1: Definition of the variables for the leg kinematics and gait patterns and examples for the factorial design experiments (A, B, C) and the glass beads experiments (D, E, F) . (A) Definition of the five leg variables on the factorial design experiments. Stride length, represented by the black continuous line on image 3, is the distance traveled between two consecutive contact events with the surface (images 1 and 3). Slip length, represented by the red dotted line on image 3, is the distance traveled between the beginning of a stance phase and the beginning of the next swing phase (images 1 and 2). It is non-null only when the ants are slipping backwards. Swing length, represented by the blue dashed line on image 3, is the distance traveled between the beginning of a swing phase and the beginning of the next stance phase (images 2 and 3). (B, C) Examples of locomotory patterns in the solid-flat-smooth (B) and pit (C) treatments. The time is in abscissa and the two examples have the same duration. The legs in stance and swing phases are in white and black respectively. The right (R1-R3, from anterior to posterior) and left (L1-L3) legs are shown from bottom to top. The chosen ants are located nearest to the center of their corresponding treatment in the principal component analysis in Figure 2-B. They correspond to the movies 1 and 2 (see supplementary material). (D): Set-up used for measuring the ant's trajectories on an inclined plane at angle θ. (E) Zoom on a part of one picture. The light pinked zone is the ant body as extracted from image analysis. The green cross is the body center, the red arrow is the oriented (from body to head) direction of the body, and the blue arrow is the direction vs of the smoothed velocity. Angles α and β define respectively the velocity and body axis inclinations with respect to the upward direction. (F) The green curve is an example of a trajectory with some pictures of the ant added.

Biological material

For the glass bead experiments we used workers of the woodland ant *Aphaenogaster subterranea* (Latreille 1798) (Hymenoptera: Formicidae), coming from five colonies located on the campus of Beaulieu in Rennes (France, 48°6' N 1°38' W). Ants were used for experiments the same day of their capture.

Experimental design

The set-up of the second series of experiments is presented on Figure 1-D. In these experiments we used glass beads with three different mean diameters d = 500, 250 and 180 μm with a typical polydispersity (variation with respect to the mean diameter value) for each size of ± 20%. First, a bed of glass beads was prepared by pouring the granular material into a box (length = 22 cm, width = 14 cm, height = 5 cm). A layer of glass beads was previously glued on the bottom and on the sides of the box to prevent the slippage of the granular material. The granular surface was then leveled. The solid packing fraction obtained with this preparation is the "critical state" volume fraction, which is = 0.60 for quasi-monodisperse beads [22]. The box was slowly inclined-up to an angle θ. Once the inclined surface is ready, an ant was gently put at the center of the surface. The ants trajectories on the inclined surface were filmed by means of a *Photron* SA3VM2 camera with a *Nikon* 50 mm F1.8 DAF lens at 125 frames per second and a 1024 x 1024 pixel resolution. Films were stopped as soon as the ant disappeared from the field of the camera. For one inclination and one bead diameter d, between 7 and 12 ants are recorded with one trajectory per ant (see Figure 1-D). After 5 or 6 trajectories, the avalanche angle was measured. Each experimental condition (inclination + bead diameter) was repeated twice. During experiments the room hygrometry was kept between 40 and 45% and we checked that no systematic variations of the avalanche angle occurred from day to day.

Data acquisition and analysis

Depending on the granulometry and on the inclination, ants exhibit more or less difficulty to move. In order to quantify this apparent difficulty, we introduce an abnormality index which is determined through the analysis of the position and orientation of the ants during locomotion. The position of the ant [x(t), y(t)] was represented by the center of its body (green cross in the Figure 1-E), while the orientation was determined by the angle β, which describes the tilt of the main axis of the body (with positive direction towards the head, see Figure 1-E)) with respect to the vertical direction. We also defined an angle $\alpha = \arctan(dy_s/dx_s)$, that measures the inclination of velocity with respect to the vertical direction (blue arrow in Figure 1-E). The image treatment used to estimate the positions and orientations is described in Appendix A.1.

The difference of angles $\alpha - \beta$ represents the non coaxiality of the two directions. Because of the circularity of the angle, we compute this difference as $f(\beta - \alpha)$ with $f(x) = |((x + \pi) mod\, 2\pi) - \pi|$. This definition of the difference is such that $f(0) = 0 \leq f(x) \leq f(\pi) = \pi$. For a trajectory where velocity and body axis are uncorrelated, the average of the disorder parameter is $< f(\beta - \alpha) >= \pi/2$, where $< \cdot >$ represents the average value on the trajectory. We define a "walk abnormality index" for an inclination and a bead diameter d as:

$$I = \frac{1}{\sum_i t_i} \sum_i \int_0^{t_i} f(\beta(t) - \alpha(t)) w(\alpha(t)) dt. \quad (1)$$

The summation over i designates a summation over all the trajectories for a given inclination θ and grain size d, and t_i is the duration of the ith trajectory. The function $w(\alpha)$ is a positive weighting function which is maximum around $\alpha = \pi$, i.e. when the ant's velocity is in the down direction, and zero otherwise. We used $w(\alpha) = 1/\delta\alpha$ if $\pi - \delta\alpha/2 < \alpha < \pi + \delta\alpha/2$, and $w(\alpha) = 0$ elsewhere. This weighting highlights the part of the trajectory where the ants slide down in the

computation of non-coaxiality. We used $\delta\alpha = \pi/2$ in the following. Changing the values of the smoothing time τ_s used for the trajectory interpolation (see Appendix A.1) and of $\delta\alpha$ does not modify the trends of the variation of I with repsect to d and θ.

Identification of falls

The probability to fall was determined by studying the trajectory of ants on the y-axis. For each ant, its position was first smoothed out using non-weighted moving averaging on 13 images (0.1 s) to decrease noise. Then, the first derivative was used (i.e. speed). We identified falls as local minima on the y-axis of the trajectories. A fall had to last at least 0.5 s to be considered as such. This threshold corresponds to the mean gait period observed in the granular-inclined treatments (see 2), which means that if a downwards trajectory lasts longer than the typical gate period, the ant is considered to be falling down the slope. Using these criteria falls where identified semi-automatically by trajectory analysis and then verified visually on the film.

3 Results

In the first series of experiments with natural sand, the two granular-inclined treatments (T6&T7), gave similar results for the six variables studied: speed, stride period, duty factor, stride length, swing length and slip length considered in the world frame. The five non-(granular-inclined) treatments (T1-T5) also gave similar results among themselves. The two granular-inclined treatments (T6 & T7) gave results different from those for the other five treatments (Table 1 & Table 3, Figure 2-B and supplementary materials movies 1 and 2). Ants moved three to four times slower in the two granular-inclined treatments (T6 & T7) than in the other five treatments. In the non-(granular-inclined) treatments (T1 to T5), the legs moved almost exclusively during the swing phase, whilst during the stance phase leg movements were negligible. By contrast, backwards leg movements during the stance phase in the two granular-inclined treatments (T6 & T7) were between 2 and 4 times more than in the other five treatments. As legs moved in opposite directions during the stance and swing phases, the resulting stride length was shorter than the swing length when considered in the world frame (Figure 2-A). In the body frame, the legs also extended less in the two granular-inclined treatments (T6 & T7) than in the other five treatments (Figure 3). The difference between the two granular-inclined treatments (T6 & T7) and the other five treatments was confirmed in the body frame, but was of smaller amplitude than in the world frame. In terms of inter-leg locomotory patterns, ants mainly walked with a tripod gait in the five non-(granular-inclined) treatments. By contrast, they mainly walked with a metachronal gait (i.e. one leg in the air, instead of three) in the two granular-inclined treatments (T6 & T7) (Figure 4-A). The surface of stability is therefore considerably increased on granular slopes (Figure 4-B). Generally, increasing the number of legs in contact increases the stability surface, irrespective of the terrain (Figure 4-C). An overview taking into account the frequency of transitions between gait patterns and their occurrence shows that granular slopes are very different from other conditions: several gait patterns occur frequently, instead of only once, and ants change gait pattern frequently, leading to a stricking increase of locomotory complexity (Figure 5).

Concerning the analysis of the second series of experiments with model sand, Figure 6 shows the measures of the two angles α and β as a function of time for two different ants. Figure 6-A corresponds to a walk on a flat surface $\theta = 0$ with $d = 250$ μm . We first observe that $\alpha \sim \beta$ during the walk. This indicates that the body velocity is oriented along the body axis. We also observe some oscillations of the orientation. The peak to peak amplitudes are typically $\sim$0.5 rad which corresponds to oscillations of $\pm$ 15° at a frequency $\sim$ 1 Hz. The situation is strikingly different for an ant moving on an inclined surface with d = 250 μm (Figure 6-B). Firstly, the body oscillations are no longer visible on β, and secondly, the orientation of the ant velocity

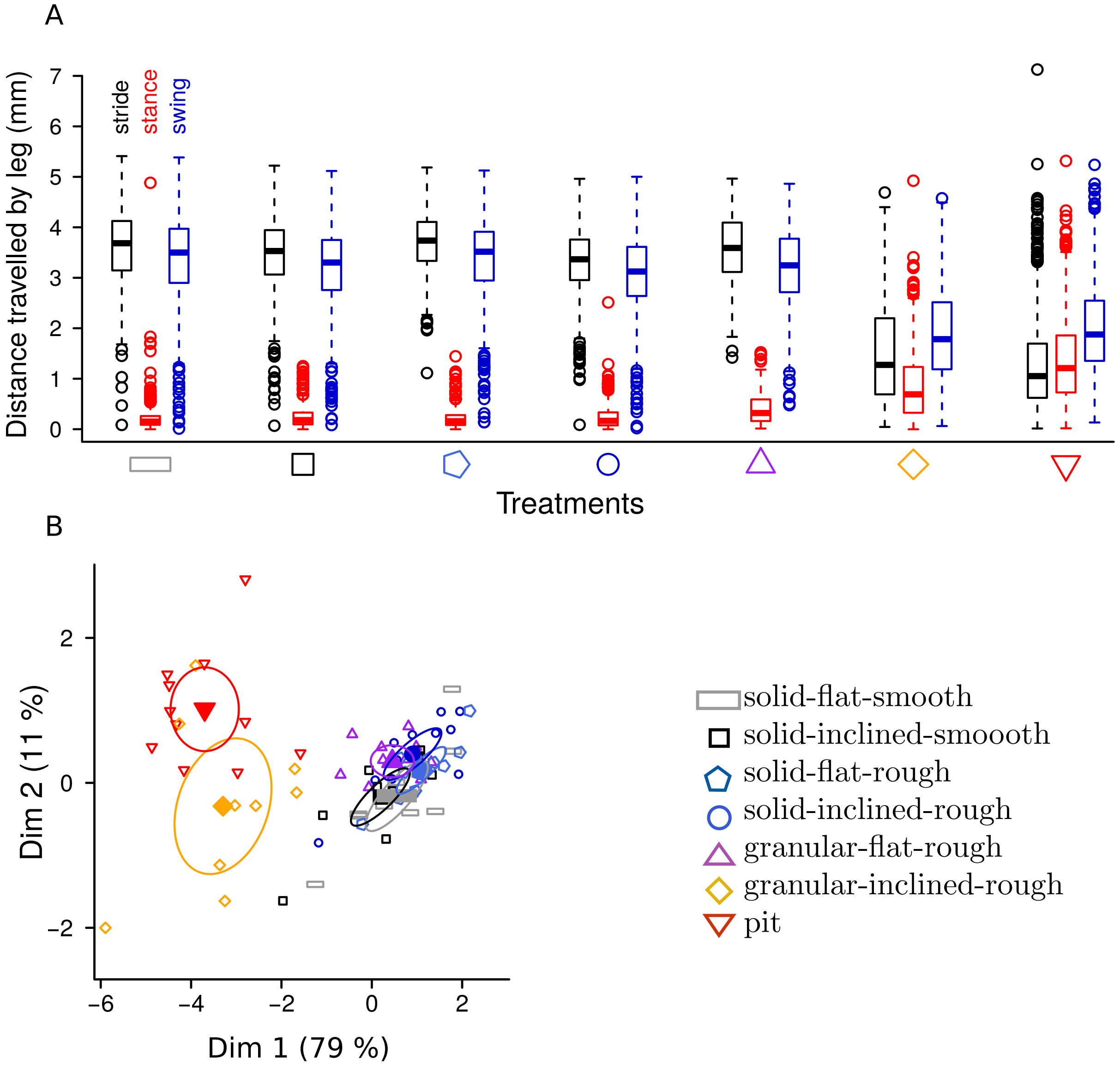

Figure 2: Leg kinematics and distance travelled in the world frame. (A) Distance travelled by legs during a stride (black, left), a stance phase (red, center) and swing phase (blue, right) as function of the treatment. Each boxplot shows the distances of all strides of the six legs of all ants for the corresponding treatment. Two points are outside the window. (B) Principal component analysis of leg kinematics in the factorial design experiments. Individuals are ants (open symbols) and the variables are the speed and five leg variables. The closed symbols are the means of the seven treatments. Ellipses indicate the 95% confidence intervals of the means. The 6 variables are all well represented using the first two axes and represent between 86 and 94% of inertia, depending on the variable.

#	T2 (sfs)	T3 (sfr)	T4 (sir)	T5 (gfr)	T6 (gir)	T7
T1 (sfs)	&Period	Speed<br>Duty	Period<br>Period<br>Duty	Stance all	all	
T2 (sis)		Speed<br>Duty	Speed<br>Period<br>Duty	Speed<br>Stance	all	all
T3 (sfr)			Swing	Duty<br>Stance	all	all
T4 (sir)				Duty<br>Stance	all	all
T5 (gfr)					all	all
T6 (gir)						Stance

Table 3: Comparison tests between treatments. We applied Mann-Whitney tests for each locomotion variable to compare two treatments. The names are those of the variables that are statistically different at the 0.05 threshold.

appears very fluctuating, and uncorrelated to the body orientation. This corresponds to an ant's locomotion where the velocity direction does not coincide with body axis anymore. The difference of angles $\alpha - \beta$ represents the non coaxiality of the two directions. This difference is very low in the case depicted on Figure 6-A corresponding to the walk on a smooth horizontal surface. In contrast, for inclined surfaces, Figure 6-B shows that this difference may be important. The walk abnormality index is presented in Figure 7 for the three granulometries, and for different values of the distance to the avalanche $\Delta\theta = \theta_a - \theta$. We observe that the index increases as the slope approaches the avalanche angle. This indicates that the coaxiality between the body axis and the velocity of the ant diminishes. This shows that our newly designed walk abnormality index is a reasonable definition of the difficulty to walk.

4 Discussion

4.1 Ant locomotion on sandy slopes is different from all other treatments

The first series of experiments shows that the locomotion of the ant *A. subterranea* in the pit and on the (artificial-granular-slope) were similar and stood out as different from all the other treatments. The differences between some of the locomotion parameters (such as the the leg extension), were larger when measured in the world frame. These results were also confirmed in the body frame, however differences between treatements were smaller. The angle of the two granular-inclined treatments differed from the other inclined treatments by being 8° less steep. This difference implies that the outcome for identical slopes would be even more pronounced than observed. The difficulty of walking in the two granular-inclined treatments translated into a change in the gait pattern, from the classical tripod gait to a metachronal gait. Each leg also spent more time on the surface, with a smaller stride amplitude. These changes are in agreement with the locomotion generally observed with a reduction in speed, on all kinds of substrates [12, 21, 23–28]. Ants may hold legs on to the surface for longer periods of time in the granular-inclined conditions to ensure stability and to avoid falling because of unpredictable reactions of the medium [25, 29, 30]. Also, the disappearance of lateral body oscillations in the two granular-inclined treatments can be interpreted as an attempt to increase stability. This kind of behavior at low speeds has been studied before in the theoretical modelling of the locomotion of cockroaches in horizontal and inclined planes [31, 32].

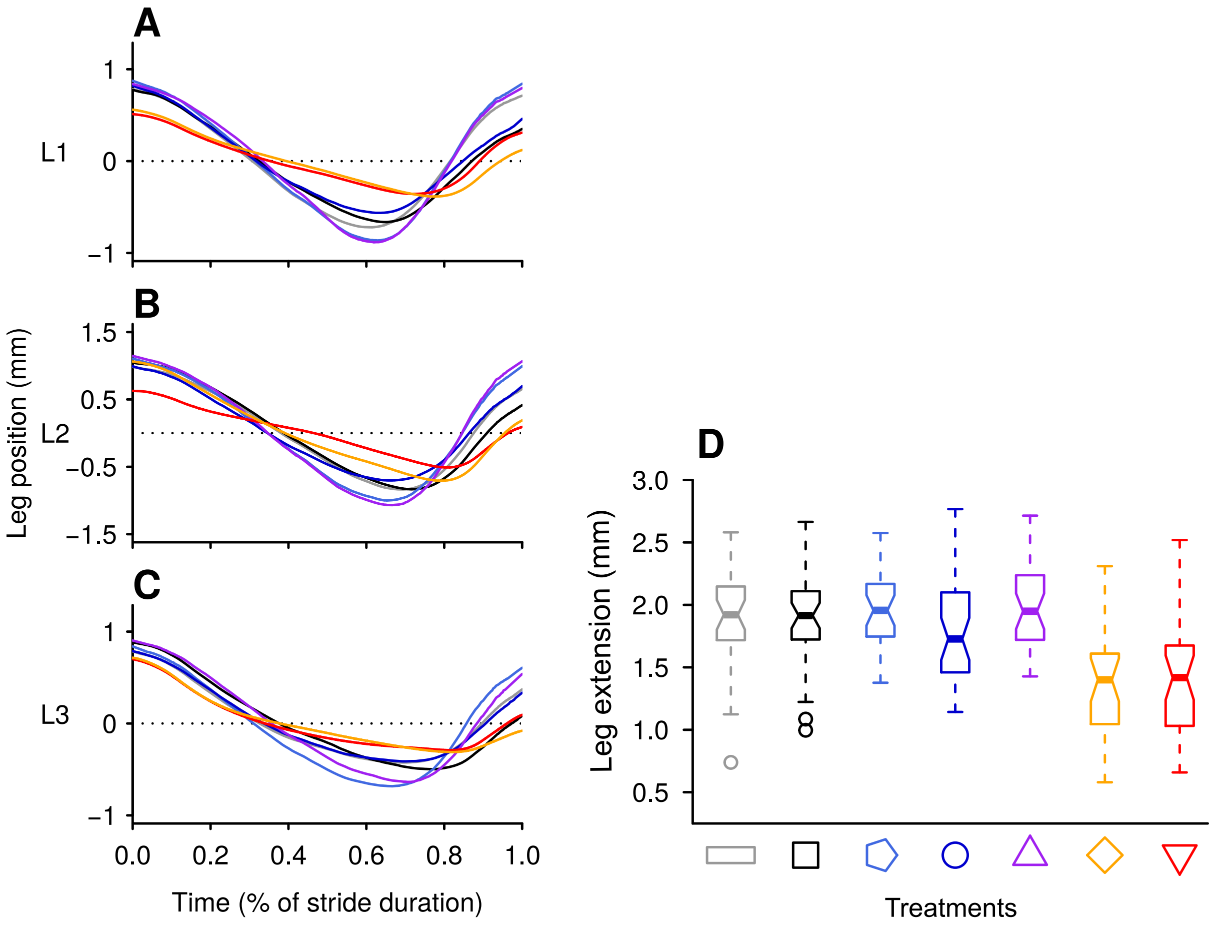

Figure 3: Leg kinematics and distance travelled in the ant frame. Leg trajectories during a stride for each treatment of the factorial design experiments for the front (A), the middle (B) and the hind (C) legs and the extension of legs in the ant frame (D). Time starts with the beginning of a stance and finishes with the end of the swing phase. In order to compare trajectories of the three pairs of legs, which have different positions in the antero-posterior axis, each trajectory is centered on its mean position during a stride. (D) Each boxplot includes the front, middle and hind legs for all ants of the corresponding treatment. See figure 3 for the explanation of the symbols representing the different treatments.

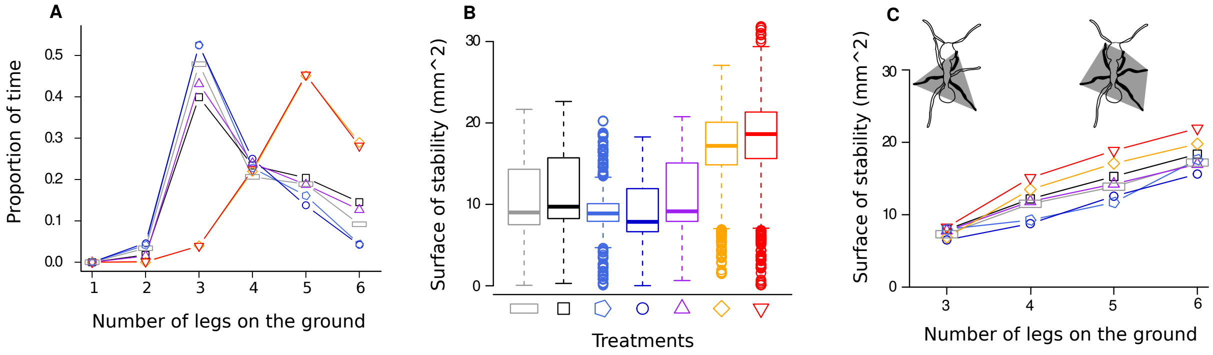

Figure 4: Ant stability for the different treatments. (A) Proportion of time spent with different number of legs on the ground of the factorial design experiments. Each line is the mean value of all ants recordings per treatment. (B) Surface of stability polygon in mm^2. Each boxplot includes the front, middle and hind legs for all ants of the corresponding treatment. (C) Mean surface of stability as function of the number of legs on the ground. The surface of stability is sketched in grey for the cases of 3 and 5 legs on the ground. See figure 3 for the explanation of the symbols representing the different treatments.

The most important result is the backward leg movements during the stance phase observed in the two granular-inclined treatments, leading to slipping. Indeed, it is an atypical locomotion pattern, observed generally on slippery surfaces [25, 33–35]. Slipping is the inability to maintain limb contact stability and is due to low frictional forces between leg and ground [36]. How does slipping come about on a granular medium? Slope, without the interaction with sand, had no effect on the locomotion of *A. subterranea*, either in terms of body speed, leg variables or gait. Our results are thus consistent with previous work observing little impact of slope on insect locomotion in general [25, 37–41]. Slope alone is thus not sufficient to explain the struggling of ants on sandy slopes. Furthermore, the locomotion of *A. subterranea* was similar on smooth and rough surfaces simulated by glued sand grains, a result again consistent with previous work observing easy locomotion both on smooth and on rough surfaces (see for example [26, 39, 42–44] for changes in speed and sinuosity). Finally, the locomotion of *A. subterranea* was similar on granular-flat and solid media. Our results thus show that neither the granular nature nor the roughness properties of sand alone are sufficient to explain the struggling of ants on sandy slopes: the interaction between the sand properties and the slope are key.

We cannot explain the backward leg movements observed in the pit and on a sandy slope by specific friction properties of the granular material. Indeed, we would in that case expect an effect of roughness too, namely a difference between the solid-inclined-smooth treatment and the two inclined-rough treatments, solid and granular. Such difference was not observed. Instead, backward movements of legs seem to be related to localised movements of sand just under the leg, as if each leg was positioned on an localised treadmill. We recently established a theory which explains the observed localized behavior [18], using inert objects of known properties and friction coefficients sliding down an inclined sand slope as a further way to control the experimental conditions. Briefly, sliding occurs around a precise value of the pressure exerted by the object on the substrate, and the estimated value for *A. subterranea* is close to it. No erosion occurs at smaller pressures, so the sand acts then as a solid substrate. At higher pressures, the object produces a frontal bulge which stops it after a small displacement. Our results obtained in the second set of experiments using glass beads confirm the latter. The abnormality index plot presented in Figure 7 is strikingly similar to the sliding probability plot for inert objects on sandy slopes shown in Figure 3-a in [18]. Indeed, the abnormality index puts into evidence the local slipping of the granular media as a consequence of the pressure exerted by the ant's legs. The presence of a more important tangential component of the stress makes locomotion

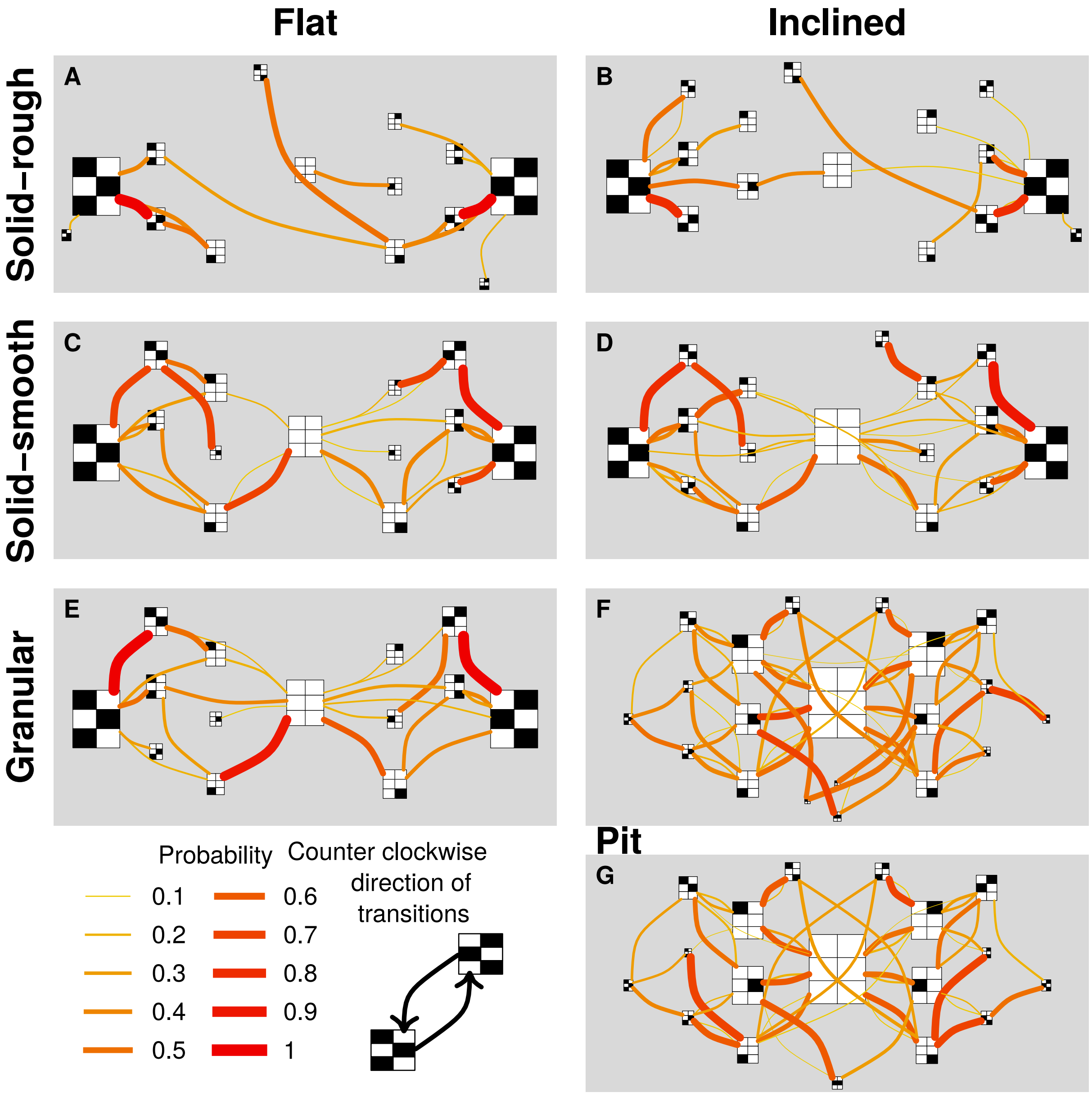

Figure 5: Ethograms of leg configurations by treatment. Legs are either in stance or in swing state represented by white or black blocks respectively. The head is pointing upwards. The surface of each rectangle (composed of 6 wight or black blocks representing the legs) is proportional to the observed frequency of each gate pattern (i.e. bigger rectangles mean a bigger time spent by ants performing that type of gate pattern). The lines between different gate patterns show the probability to shift from one to the other. Linewidths indicate higher or lower probabilities (see figure legend) and the direction of the transitions should be considered counter clockwise according to the line curvatures. Gate pattern transitions are indicated only if at least five transitions were observed and if the probability was higher than 5%.

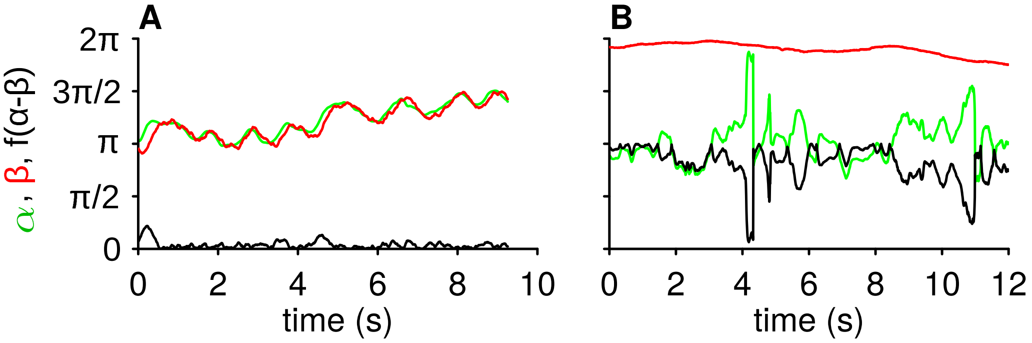

Figure 6: Examples of ant trajectories on flat (A) and inclined (B) planes of glass beads. Angles in radians of the angles α (green curve), β (red curve) and $f(\alpha-\beta)$ (black curve) as the function of the time. $f(x) = |(x+\pi)mod2\pi - \pi|$. (A) The inclination is $\theta = 0$ deg and bead diameter $d = 250\mu m$. (B) The inclination is $\theta = 27$ deg and bead diameter $d = 250\mu m$

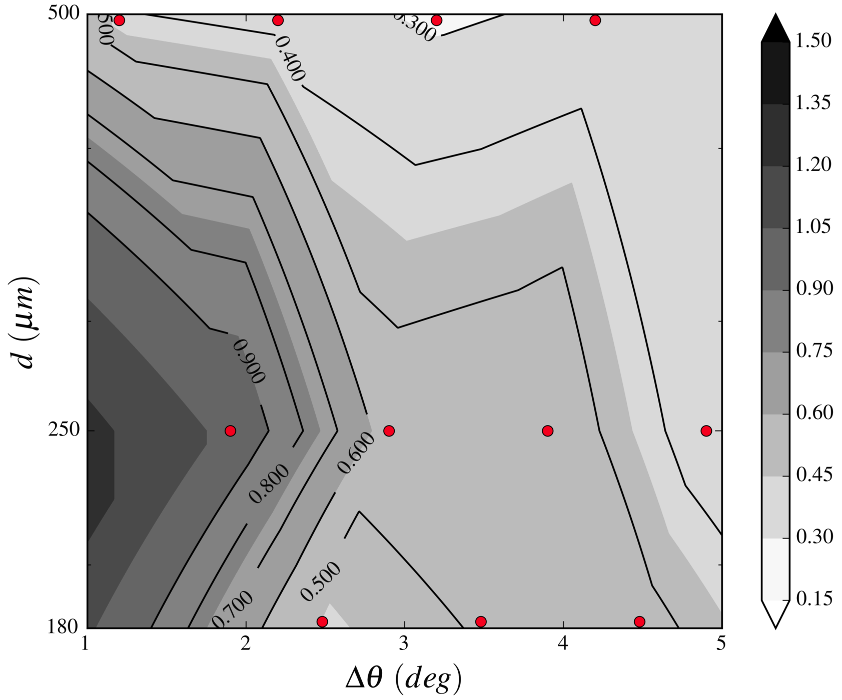

Figure 7: Walk abnormality index as function of slope and glass bead diameter. The grain diameter is logarithmic. The construction of the map is made with linear interpolations of measured I values (red dots) and considering an abnormality index $I = \pi/2$ for all grain sizes at $\Delta\theta = 0$.

on slope different from locomotion on flat surfaces. This understanding opens new venues for relating the pressure exerted on a substrate by insects and their locomotion. This mechanism is operating in the antlion's pit and maybe in other contexts, as described next.

4.2 Restricted conditions for slipping

The second series of experiments shows that, near to the avalanche threshold, ants display a strong walk abnormality index, do slide considerably, but do not create avalanches. This observation is in agreement with the expected result that inclinations of a surface make the ant's walk more difficult, as described earlier, confirming the results of Botz *et al.* [15]. The second observation is that the index depends on the bead's size. This shows that the locomotion of insects on sand cannot be understood only in term of differences between actual and avalanche angles, and that there is some finite size effects in the physics of the locomotion which need to be considered. Finally, the most difficult walk is observed for an intermediate granulometry, i.e. the size effect is not trivial. Although we considered locomotion on an inclined surface near avalanche angle, we systematically observed that the perturbation which is created by the ant stays very local. We therefore confirm and qualify the results of Botz *et al.* [15] who observed that avalanches do not cause the ant loss of stability. However, we do not confirm that ants produce avalanche by their falling. In general, the size of the perturbed area on the granular surface is small compared to the entire experimental surface. Sand movements are in fact of small extent, the disturbed area being often as wide as long, and of ca. 10 grains each side. So, at the scale of the granular box, there is no variation of the slope of the granular material. In other words, the difficulty to move does not seem related to the occurrence of macroscopic avalanches, but only to the ability of the material to flow in response to additional stress.

Falls are the second most frequent failure mode of stability, after sliding. Falls usually were side-way rolling over or stalling, and stop after one or a couple of loops. This is another difference with the results of Botz *et al.* [15], who report that ants fall down the entire slope. However, this discrepancy maybe explained by the différence in weight between the ants used in both works. Botz *et al.* observed falls through the entire slope for ants of the species *Camponotus* with mean weight ~ 4.5 mg, whilst in our experiments the ants mean weight is ~ 1.74. The number of falls we observed was strongly function of the length of recording and therefore somewhat difficult to put into perspective. We observed 60 falls for 131 ants and a total recording time of over 30 minutes. The rate of falling is therefore one per 30 seconds, which is also about the time an ant needs to escape from an antlion pit [13].

4.3 Implications for animal and plant traps

The interaction between the granular properties of the sand and the slope of the pit creates the proper conditions for slipping of struggling prey, of a narrow range of sizes and hence weights, in antlion pits [13]. Large prey do escape out of the pit without walking difficulties by creating a rim at each step; the footprints remain visible after the prey left the pit [18]. Our experiments confirm therefore earlier measurements on slopes of antlion pits [16] showing that building a pit at a slope near the avalanche threshold is key, as it greatly increases the range of prey sizes which do struggle moving up the pit and hence the capture rate. Our findings have generality beyond the antlion pits. The nearest mechanical trapping system analogous to the antlion pit might be the urn of pitchers plants that use slippery surfaces to capture insects, especially in the genus *Nepenthes* [45, 46]. They use a surprising diversity of mechanisms to create slippery conditions [47, 48] in order to orient the prey towards the deadly part of the plant (see for example [49]). Epicuticular wax crystals can decrease adhesion by contaminating tarsi of the prey [46, 50, 51]. Short video sequences of the ant *Polyrhachis pruinosa* trying to move up the waxy *N. hemsleyana* urns (video courtesy L. Gaume) display a locomotory pattern very similar to the one-swing gait described in our study: the hind legs are dragged, the mid-legs do all the

work, and the front legs are searching for anchoring points. Hind legs dragging is a pattern which is interpreted as enhancing stability [9]. The switch of functional roles of legs from pushing to pulling is also a typical characteristics of locomotion on inclines [32, 39]. In the case of granular inclines, local slipping may play an essential role in the locomotory pattern transitions. Recent studies have shown the importance of load mechanisms in the leg coordination of insects [52–54]. The sudden lose of traction due to the gliding of grains can generate a reduction of leg loading, and so triggering a gait transition.

Our results also shed light on other situations where sliding might occur, and where stability is certainly at risk. Some ants build conical sand structure at the entrance of their nest and they walk normally on their slope, including when the slope is the slope of avalanches or the angle of repose [11, 12, 55]. This is a puzzling behavior. One possible explanation was given above, related to the mismatch between the pressure of legs and the size of a grain. Two studies on different ant species and locations report the diameter of the sand particles: over 1 mm in both cases [55, 56]. This is a very large value for a sand grain, about one order of magnitude larger than what we used, for ants which are not larger. Alternatively, the explanations lie rather with the substrate. Indeed, ants might rather have transported a pellet of cohesive grains, either due to buccal fluids and colloidal substances, or because these nests are often excavated after heavy rains, when excavation is possible [12]. In such case, the cohesive forces might stabilize the heap and enable ants to move up the nest slopes without difficulties (note that larger ants seem to have difficulties, [12]). The properties of the granular structures may also depend on the preparation's history. This is the case for sand pile built by ants [12], where grains are deposited near the bottom of the heap, and may stop or roll down. The building method and the fluctuations of the rolling activity are reminiscent of the sandpile building method used for physical models of Self Organized Criticality [57]. We lack more comprehensive descriptions of leg kinematics for struggling insects on different surfaces to deepen the analogy with our work, beyond the recurrent observation of an increase tendency of ants to gain stability by increasing the number of legs in contact with the slippery substrate.

5 Conclusions

Our results pinpoint towards generic trade-offs small insects face when striving for stability on unstable terrain [9]. The number of legs in contact can be increased to promote their stability, by increasing the overall surface of contact. This is what is observed on ants and also on larger animals and efficient robots [58–60]. Alternatively, the number of legs in contact with the substrate can be decreased, in order to increase the weight supported by each leg and hence the penetrating ability, thereby creating a rim [61]. The first strategy decreases the pressure exerted on the substrate by each leg, and might lead to sliding. The second strategy implies a small number of legs in contact with the substrate, and this might lead to a loss of stability and stumbling. Creating a rim for a light species is difficult and the trade-off therefore specific to light, small animals. Large animals and robots face a different trade-off as the second strategy leads to fluidization, and hence a sinking in of the animal or robot [59]. In these trade-offs, the pressure exerted on the substrate is key, and thus is the body weight and its distribution among legs. According to our recordings, a struggling ant foot is in contact with about one, at most ten, sand grains at each step. How static and dynamic body stabilities are managed by small insects walking on rugged terrain [7, 9] will thus require a thorough understanding of the transfer of energy from leg to sand, at the grain level.

A Appendix

A.1 Position and orientation estimation

We describe here the algorithm for the estimation of the ants' position and orientation. It is an automation on R (R Core Team 2013) of the principles using in the sand experiment (see Data acquisition). The set of three values (contrast, median filter and binary threshold) were visually determined for $\approx 2\%$ of the 621 496 images and automatically determined for others. The algorithm used to choose the set of values has been independently applied on each ant. First we apply a manual step: 10 images (the first, the last and 8 with regular span) were manually selected with visual determination of the set of values (contrast, median filter and binary threshold). A first automatic step is then applied: all sets of values were applied on all images non-previously selected. We chose one set of values for each frame based on the surface of the selection and on the mean surface of the ants manually selected. We finally determined if this set fitted well enough the ant or not. We considered the selection as satisfying if the area of the selection was within the range area of manually selected ants. In the second manual step we manually selected the 5 worse non satisfying images. In the second automatic step we applied the automatic step with the new set of values. Other manual and automatic steps were executed until all images were properly selected.

Subsequent to image analysis data is processed as follows : First, we remove some experimental noise on the ant's position $(x(t), y(t))$. For this, the functions $x(t)$ and $y(t)$ are approximated for every t by a smoothed position $(x_s(t), y_s(t))$. Therefore we have interpolated $x(t)$ and $y(t)$ with two second order interpolation polynomials of second degree. Interpolation is done between times $t - \tau_s$ and $t + \tau_s$ with a smoothing time $\tau_s = 0.1$ s. Let $v_s(t) = (dx_s/dt, dy_s/dt)$ the smoothed velocity.

A.2 Spatial calibration

The data were transformed from the horizontal plane to the two-dimensional plane of locomotion, and from pixels to millimetres. A grid of black squares (0.25 ± 0.03 mm^2) printed on white paper was placed on the surface of the medium. It was glued to a flat surface before being placed on the surface of the medium, except for the pit, in which it was manually curved to fit the pit. The coordinates of the centres of the squares were selected as described for the coordinates of the ant's body, but without the median filter (see Data Acquisition). The mean distance between the centers of two neighboring squares (1.00$\pm$ 0.03 mm) was between 43 and 91 pixels/mm, depending on the film. Image resolution thus varied between 11 and 23 pixels/mm. In inclined treatments (T2,T4,T6,T7), the distance between the surface and the camera varied, resulting in a variation of resolution around the mean. We thus applied a position-dependent correction. For each film, the standard deviation of the distance provided information about data accuracy. This accuracy was systematically better than 0.10 mm, the diameter of the smallest sand grain.

References

[1] Edwards J. S. 1987. Arthropods of alpine aeolian ecosystems. *Annual Review of Entomology*, 32:163–179.

[2] Glover A. G., Gooday A. J., Bailey D. M., Billett D. S. M., Chevaldonne P., Colaco A., Copley J., Cuvelier D., Desbruyeres D., Kalogeropoulou V., Klages M., Lampadariou N., Lejeusne C., Mestre N. C., Paterson G. L. J., Perez T., Ruhl H., Sarrazin J., Soltwedel T., Soto E. H., Thatje S., Tselepides A., Van Gaever S., and Vanreusel A. Temporal change in deep-sea benthic ecosystems: A review of the evidence from recent time-series studies. In

Lesser, M, editor, *Advances in Marine Biology, Vol 58*, volume 58 of *Advances in Marine Biology*, pages 1–95. 2010.

[3] Eigenbrode S. D., Snyder W. E., Clevenger G., Ding H., and Gorb S. N. *Variable Attachment to Plant Surface Waxes by Predatory Insects*, pages 157–181. Springer Netherlands, Dordrecht, 2009.

[4] Shao C.-P., Chen Y.-J., and Lin J.-Z. 2012. Wind induced deformation and vibration of a Platanus acerifolia leaf. *Acta Mechanica Sinica*, 28(3):583–594.

[5] Bohn H. F. and Federle W. 2004. Insect aquaplaning: Nepenthes pitcher plants capture prey with the peristome, a fully wettable water-lubricated anisotropic surface. *Proceedings of the National Academy of Sciences*, 101(39):14138–14143.

[6] Hansell M. H. *Animal architecture*. Oxford animal biology series. Oxford University Press, 2005.

[7] Li C., Pullin A. O., Haldane D. W., Lam H. K., Fearing R. S., and Full R. J. 2015. Terradynamically streamlined shapes in animals and robots enhance traversability through densely cluttered terrain. *Bioinspiration & Biomimetics*, 10(4).

[8] Jayaram K. and Full R. J. 2016. Cockroaches traverse crevices, crawl rapidly in confined spaces, and inspire a soft, legged robot. *Proceedings of the National Academy of Sciences of the United States of America*, 113(8):E950–E957.

[9] Weihmann T., Goetzke H. H., and Guenther M. 2015. Requirements and limits of anatomy-based predictions of locomotion in terrestrial arthropods with emphasis on arachnids. *Journal of Paleontology*, 89(6):980–990.

[10] Smolka J., Byrne M. J., Scholtz C. H., and Dacke M. 2013. A new galloping gait in an insect. *Current Biology*, 23(20):R913–R915.

[11] Franks N. R., Britton N. F., Sendova-Franks A. B., Denny A. J., Soans E., Brown A., Cole R., Havardi R., Griffiths C., and Ellis S. 2004. Centrifugal waste disposal and the optimization of ant nest craters. *Animal Behaviour*, 67(5):965–973.

[12] Tofilski A. and Ratnieks F. L. 2005. Sand pile formation in Dorymyrmex ants. *Journal of Insect Behavior*, 18(4):505–512.

[13] Humeau A., Rouge J., and Casas J. 2015. Optimal range of prey size for antlions. *Ecological Entomology*, 40(6):776–781.

[14] Lucas J. R. 1982. The biophysics of pit construction by antlion larvae (Myrmeleon, neuroptera). *Animal Behaviour*, 30(AUG):651–&.

[15] Botz J. T., Loudon C., Barger J. B., Olafsen J. S., and Steeples D. W. 2003. Effects of slope and particle size on ant locomotion: Implications for choice of substrate by antlions. *Journal of the Kansas Entomological Society*, 76(3):426–435.

[16] Fertin A. and Casas J. 2006. Efficiency of antlion trap construction. *Journal of Experimental Biology*, 209(18):3510–3515.

[17] Forterre Y. and Pouliquen O. 2008. Flows of dense granular media. *Annual Review of Fluid Mechanics*, 40:1–24.

[18] Crassous J., Humeau A., Boury S., and Casas J. 2017. Pressure-Dependent Friction on Granular Slopes Close to Avalanche. *Physical Review Letters*, 119(5).

[19] Bocquet L., Charlaix E., Ciliberto S., and Crassous J. 1998. Moisture-induced ageing in granular media and the kinetics of capillary condensation. *Nature*, 396(6713):735–737.

[20] Fertin A. and Casas J. 2007. Orientation towards prey in antlions: efficient use of wave propagation in sand. *Journal of Experimental Biology*, 210(19):3337–3343.

[21] Holmes P., Full R. J., Koditschek D., and Guckenheimer J. 2006. The dynamics of legged locomotion: Models, analyses, and challenges. *SIAM Review*, 48(2):207–304.

[22] Gravish N., Umbanhowar P. B., and Goldman D. I. 2014. Force and flow at the onset of drag in plowed granular media. *Physical Review E*, 89(4).

[23] Wilson D. M. 1966. Insect walking. *Annual Review of Entomology*, 11:103+.

[24] Kram R., Wong B., and Full R. J. 1997. Three-dimensional kinematics and limb kinetic energy of running cockroaches. *Journal Of Experimental Biology*, 200(13):1919–1929.

[25] Seidl T. and Wehner R. 2008. Walking on inclines: how do desert ants monitor slope and step length. *Frontiers in Zoology*, 5.

[26] Bernadou A. and Fourcassié V. 2008. Does substrate coarseness matter for foraging ants? an experiment with lasius niger (hymenoptera; formicidae). *Journal of Insect Physiology*, 54(3):534–542.

[27] Reinhardt L. and Blickhan R. 2014. Level locomotion in wood ants: evidence for grounded running. *Journal of Experimental Biology*, 217(13):2358–2370.

[28] Khuong A., Lecheval V., Fournier R., Blanco S., Weitz S., Bezian J.-J., and Gautrais J. 2013. How Do Ants Make Sense of Gravity? A Boltzmann Walker Analysis of Lasius niger Trajectories on Various Inclines. *PLOS ONE*, 8(10).

[29] Ting L. H., Blickhan R., and Full R. J. 1994. Dynamic and static stability in hexapedal runners. *Journal of Experimental Biology*, 197:251–269.

[30] Voloshina A. S., Kuo A. D., Daley M. A., and Ferris D. P. 2013. Biomechanics and energetics of walking on uneven terrain. *Journal of Experimental Biology*, 216(21):3963–3970.

[31] Schmitt J., Garcia M., Razo R., Holmes P., and Full R. J. 2002. Dynamics and stability of legged locomotion in the horizontal plane: a test case using insects. *Biological Cybernetics*, 86(5):343–353.

[32] Schmitt J. and Bonnono S. 2009. Dynamics and stability of lateral plane locomotion on inclines. *Journal of Theoretical Biology*, 261(4):598–609.

[33] Epstein S. and Graham D. 1983. Behavior and motor output of stick insects walking on a slippery surface .1. forward walking. *Journal of Experimental Biology*, 105(JUL):215–229.

[34] Brady R. A., Pavol M. J., Owings T. M., and Grabiner M. D. 2000. Foot displacement but not velocity predicts the outcome of a slip induced in young subjects while walking. *Journal of Biomechanics*, 33(7):803–808.

[35] You J.-Y., Chou Y.-L., Lin C.-J., and Su F.-C. 2001. Effect of slip on movement of body center of mass relative to base of support. *Clinical Biomechanics*, 16(2):167–173.

[36] Clark A. J. and Higham T. E. 2011. Slipping, sliding and stability: locomotor strategies for overcoming low-friction surfaces. *Journal of Experimental Biology*, 214(8):1369–1378.

[37] Wohlgemuth S., Ronacher B., and Wehner R. 2002. Distance estimation in the third dimension in desert ants. *Journal of Comparative Physiology A-Neuroethology Sensory Neural and Behavioral Physiology*, 188(4):273–281.

[38] Lipp A., Wolf H., and Lehmann F.-O. 2005. Walking on inclines: energetics of locomotion in the ant Camponotus. *Journal of Experimental Biology*, 208(4):707–719.

[39] Goldman D. I., Chen T. S., Dudek D. M., and Full R. J. 2006. Dynamics of rapid vertical climbing in cockroaches reveals a template. *Journal of Experimental Biology*, 209(15):2990–3000.

[40] Weihmann T. and Blickhan R. 2009. Comparing inclined locomotion in a ground-living and a climbing ant species: sagittal plane kinematics. *Journal of Comparative Physiology A-Neuroethology Sensory Neural and Behavioral Physiology*, 195(11):1011–1020.

[41] Holt N. C. and Askew G. N. 2012. Locomotion on a slope in leaf-cutter ants: metabolic energy use, behavioural adaptations and the implications for route selection on hilly terrain. *Journal of Experimental Biology*, 215(15):2545–2550.

[42] Walker G. 1993. Adhesion to smooth surfaces by insects - A review. *International Journal of Adhesion and Adhesives*, 13(1):3–7.

[43] Dai Z., Gorb S. N., and Schwarz U. 2002. Roughness-dependent friction force of the tarsal claw system in the beetle Pachnoda marginata (Coleoptera, Scarabaeidae). *Journal Of Experimental Biology*, 205(16):2479–2488.

[44] Labonte D. and Federle W. 2015. Scaling and biomechanics of surface attachment in climbing animals. *Philosophical Transactions of the Royal Society B-Biological Sciences*, 370(1661).

[45] Riedel M., Eichner A., and Jetter R. 2003. Slippery surfaces of carnivorous plants: composition of epicuticular wax crystals in Nepenthes alata Blanco pitchers. *Planta*, 218(1):87–97.

[46] Gaume L., Perret P., Gorb E., Gorb S., Labat J.-J., and Rowe N. 2004. How do plant waxes cause flies to slide? Experimental tests of wax-based trapping mechanisms in three pitfall carnivorous plants. *Arthropod Structure & Development*, 33(1):103–111.

[47] Bonhomme V., Pelloux-Prayer H., Jousselin E., Forterre Y., Labat J.-J., and Gaume L. 2011. Slippery or sticky? functional diversity in the trapping strategy ofnepenthescarnivorous plants. *New Phytologist*, 191(2):545–554.

[48] Gaume L., Bazile V., Huguin M., and Bonhomme V. 2016. Different pitcher shapes and trapping syndromes explain resource partitioning in Nepenthes species. *Ecology and Evolution*, 6(5):1378–1392.

[49] Gorb E. V. and Gorb S. N. Functional Surfaces in the Pitcher of the Carnivorous Plant Nepenthes alata: A Cryo-Sem Approach. In Gorb, SN, editor, *Functional surfaces in biology: Adhesion related phenomena, Vol 2*, pages 205–238. 2009.

[50] Gaume L., Gorb S., and Rowe N. 2002. Function of epidermal surfaces in the trapping efficiency of Nepenthes alata pitchers. *New Phytologist*, 156(3):479–489.

[51] Gorb E. V., Purtov J., and Gorb S. N. 2014. Adhesion force measurements on the two wax layers of the waxy zone in Nepenthes alata pitchers. *Scientific Reports*, 4.

[52] Dürr V., Theunissen L. M., Dallmann C. J., Hoinville T., and Schmitz J. 2017. Motor flexibility in insects: adaptive coordination of limbs in locomotion and near-range exploration. *Behavioral Ecology and Sociobiology*, 72(1):15.

[53] Dallmann C. J., Hoinville T., Duerr V., and Schmitz J. 2017. A load-based mechanism for inter-leg coordination in insects. *Proceedings of the Royal Society B-Biological Sciences*, 284(1868).

[54] Weihmann T. 2018. Leg force interference in polypedal locomotion. *Science Advances*, 4(9).

[55] Robinson E. J. H., Holcombe M., and Ratnieks F. L. W. 2008. The organization of soil disposal by ants. *Animal Behaviour*, 75(4):1389–1399.

[56] Tofilski A. and Ratnieks F. L. W. 2008. Simple rules based on pile slope are used in the self organization of sand pile formation by Pheidole oxyops ants. *Insectes Sociaux*, 55(1):37–42.

[57] Held G., Solina D., Keane D., Haag W., Horn P., and Grinstein G. 1990. Experimental-study of critical-mass fluctuations in an evolving sandpile. *Physical Revew Letters*, 65(9):1120–1123.

[58] Ritzmann R. E., Quinn R. D., and Fischer M. S. 2004. Convergent evolution and locomotion through complex terrain by insects, vertebrates and robots. *Arthropod Structure & Development*, 33(3):361–379.

[59] Li C., Umbanhowar P. B., Komsuoglu H., Koditschek D. E., and Goldman D. I. 2009. Sensitive dependence of the motion of a legged robot on granular media. *Proceedings of the National Academy of Sciences of the United States of America*, 106(9):3029–3034.

[60] Marvi H., Gong C., Gravish N., Astley H., Travers M., Hatton R. L., Mendelson, III J. R., Choset H., Hu D. L., and Goldman D. I. 2014. Sidewinding with minimal slip: Snake and robot ascent of sandy slopes. *Science*, 346(6206):224–229.

[61] Mazouchova N., Gravish N., Savu A., and Goldman D. I. 2010. Utilization of granular solidification during terrestrial locomotion of hatchling sea turtles. *Biology Letters*, 6(3):398–401.
